# Long-term Immunogenicity of Hepatitis A Vaccination in Adults Receiving Immunosuppressive Therapy and Adults Living With HIV: Three-year Follow-up of a Prospective Cohort Study

**DOI:** 10.1093/ofid/ofaf457

**Published:** 2025-09-15

**Authors:** Jenny L Schnyder, Hannah M Garcia Garrido, Irma Maurer, Agnes M Harskamp, Neeltje A Kootstra, Martin P Grobusch, Abraham Goorhuis

**Affiliations:** Centre of Tropical Medicine and Travel Medicine, Department of Infectious Diseases, Division of Internal Medicine, Amsterdam UMC, University of Amsterdam, Amsterdam, The Netherlands; Centre of Tropical Medicine and Travel Medicine, Department of Infectious Diseases, Division of Internal Medicine, Amsterdam UMC, University of Amsterdam, Amsterdam, The Netherlands; Department of Experimental Immunology, Amsterdam Institute for Immunology and Infectious Diseases, Amsterdam UMC, University of Amsterdam, Amsterdam, The Netherlands; Department of Experimental Immunology, Amsterdam Institute for Immunology and Infectious Diseases, Amsterdam UMC, University of Amsterdam, Amsterdam, The Netherlands; Department of Experimental Immunology, Amsterdam Institute for Immunology and Infectious Diseases, Amsterdam UMC, University of Amsterdam, Amsterdam, The Netherlands; Centre of Tropical Medicine and Travel Medicine, Department of Infectious Diseases, Division of Internal Medicine, Amsterdam UMC, University of Amsterdam, Amsterdam, The Netherlands; Centre of Tropical Medicine and Travel Medicine, Department of Infectious Diseases, Division of Internal Medicine, Amsterdam UMC, University of Amsterdam, Amsterdam, The Netherlands

**Keywords:** autoimmune diseases, hepatitis a vaccines, HIV, immunocompromised host, immunogenicity

## Abstract

**Background:**

Hepatitis A (hepA) vaccination generates long-lasting protection against hepA in healthy adults. However, the duration of protection in immunocompromised patients (ICPs), such as people living with HIV (PLWH) and patients on immunosuppressive therapy, is uncertain.

**Methods:**

This 3-year follow-up study of a prospective cohort assessed hepA antibodies in PLWH, patients on immunosuppressive therapy, and controls after completing a full hepA vaccination series. Three years later (Y3), serum samples were collected in 88/150 (59%) of original participants. The primary outcome was the seroprotection rate (SPR) at Y3, defined as the proportion of participants with hepA antibodies ≥20 mIU/mL. Secondary outcomes included seroreversion rates, defined as the proportion of those unprotected at Y3, among those initially protected after the primary vaccination schedule, and geometric mean concentrations (GMCs) at Y3.

**Results:**

At Y3, SPRs were 87% (20/23) in PLWH, 90% (26/29) in patients on immunosuppressive monotherapy, 65% (13/20) in patients on immunosuppressive combination therapy, and 100% (16/16) in controls. Seroreversion rates were 13% (3/23) in PLWH, 10% (3/29) in patients on immunosuppressive monotherapy, 21% (4/19) in patients on immunosuppressive combination therapy, and 0% (0/16) in controls. GMCs in ICPs (41.13–70.75 mIU/mL) were significantly lower compared to controls (175.65 mIU/mL) (*P*-value = .001).

**Conclusions:**

Three years postvaccination, most ICPs remained seroprotected, but SPRs and GMCs were lower than in healthy controls, particularly in patients on combination immunosuppressive therapy. However, it remains uncertain if booster doses are necessary among those who seroreverted, as long-term protection may persist through formed cellular memory.

## Teaser

Serological hepatitis A vaccine responses are less durable in people living with HIV and patients receiving immunosuppressive therapy, especially combination therapy. However, long-term protection may persist through cellular memory, thus booster doses in those who seroreverted may not be necessary.

Hepatitis A (hepA) is an inflammatory liver disease, caused by the hepatitis A virus (HAV). The clinical spectrum varies considerably, ranging from asymptomatic infection to severe hepatitis which may progress to fulminant liver failure [1]. Transmission mainly occurs fecal-orally through ingestion of contaminated food or water or through close physical contact with an infectious individual [1]. People living in low-endemic areas are at risk of acquiring hepA when travelling to areas of intermediate or high endemicity [2], or occasionally during foodborne outbreaks, such as a recent outbreak in the Netherlands, linked to contaminated frozen blueberries [3]. When infected, those with an impaired immune system, such as people living with HIV (PLWH), are at increased risk of severe disease [4–6]. The World Health Organization (WHO) recommends vaccination of people at increased risk of exposure and those at increased risk of severe disease, including travelers and PLWH [6]. HepA vaccines are highly immunogenic in healthy adults, with near 100% seroprotection rates (SPR) after 2 doses administered at a 6-month interval, and can generate long-lasting, possibly life-long protection against symptomatic disease [6, 7]. Earlier, we communicated that hepA vaccines are immunogenic in immunocompromised populations (ICPs), such as PLWH and patients on immunosuppressive therapy, with SPRs ranging from 83% to 97% measured 2 months after primary vaccination [8, 9]. However, peak hepA antibody levels are lower than in immunocompetent individuals, suggesting a shorter duration of protection [8, 9]. Few prospective studies have assessed the long-term immunogenicity of hepA vaccination among PLWH, especially among those on combination antiretroviral therapy (cART) [10–12], and to our knowledge no prospective studies have assessed the duration of protection among patients on immunosuppressive therapy beyond 2 years after vaccination [9]. Our aim was to investigate the durability of the immune response in PLWH on cART, and patients on immunosuppressive drugs, versus immunocompetent controls, 3 years after primary hepA vaccination, and to identify possible predictors of a durable serological response.

## METHODS

### Study Population

We performed a 3 year postvaccination follow-up of our prospective single-center cohort study [8]. In the initial study (2018–2023), hepA-naïve adults (≥18 years) who visited the Amsterdam UMC with an indication for hepA vaccination were included and categorized in the following groups: (1) PLWH, (2) patients on immunosuppressive monotherapy, (3) patients on immunosuppressive combination therapy (2 or more immunosuppressive agents), and (4) controls not on immunosuppressive therapy. Participants received 2 doses of inactivated hepA vaccine (Avaxim® 0.5 mL, Havrix® 1 mL, or VAQTA adult® 1 mL) at months 0 and 6–12, or when vaccination against hepB was also indicated, 3 combined hepA/B vaccine doses (Twinrix® 1 mL) at months 0, 1, and 6–12. HAV-specific antibodies levels were measured on the day of the first (M0) and last vaccination (M6), 2 months after the first (M2), and 2 and 6 months after the last vaccination (M8 and M12), respectively. To assess boostability, an additional vaccine dose had been administered 1–5 years postvaccination among a subset of participants with impeded antibody levels [8]. All participants who completed the initial study and gave consent to approach them for future studies were asked to participate by e-mail and phoned up to 3 times. No formal sample size calculation was performed as the present study aimed to include as many former participants as possible.

### Study Procedures

Venous blood samples were drawn 3 years after the first hepA vaccination (Y3) and preserved at −80°C at the Amsterdam UMC biobank until further analysis. Data regarding additional vaccines received between M12 and Y3 were collected and added to the baseline clinical and demographical data collected during the original study, using a standardized data collection form (Castor EDC).

### Laboratory Assessments

HAV-specific antibodies (both IgM and IgG) were quantified by competitive enzyme-linked immunosorbent assay (Anti-HAV ELISA, Mediagnost®, Reutlingen, Germany). Results were expressed in milli-international units per milliliter (mIU/mL). Anti-HAV levels ≥20 mIU/mL, the lower limit of detection of the assay, were considered to signify immunity against HAV infection [6].

### Study Outcomes

The primary outcome was the SPR at Y3 defined as the proportion of vaccinated participants with an antibody level ≥20 mIU/mL [6]. Secondary outcomes were: (1) seroreversion at Y3, defined as the proportion of participants who lost seroprotection at Y3 (<20 mIU/mL), among those who were previously seroprotected at M8 (≥20 mIU/mL); (2) geometric mean anti-HAV concentrations (GMCs) at Y3; (3) median fold changes in GMCs between M8 and Y3; (4) factors associated with seroprotection at Y3 and fold changes in GMCs between M8 and Y3, among PLWH and patients on immunosuppressive therapy.

### Data Analysis

Categorical data were presented in counts and percentages and continuous data as mean and standard deviation or median and interquartile ranges, as appropriate. The normality of the distribution of the data was determined by visual inspection. Antibody concentrations were analyzed on a log-transformed scale and presented as geometric means with 95% confidence intervals (CI). Associations between potential predictors and the primary outcome among PLWH and patients on immunosuppressive therapy were estimated using univariable and multivariable logistic regression models. Association between potential predictors and fold changes in GMCs between M8 and Y3 among PLWH and patients on immunosuppressive therapy were estimated using univariable and multivariable linear regression models. Potential predictors were selected based on previous findings in the literature (Supplementary Figures 1 and 2) [8, 10, 11, 13–17]. Some patients with impeded antibody levels at M12 had received a hepA booster vaccination as part of the already mentioned earlier boostability assessment [8]. Sensitivity analyses were performed to assess whether the results would change if those patients were excluded from the analysis who had received a booster dose or who had used B-cell depleting anti-CD20 therapy, which precludes antibody formation. Data analyses were performed in IBM SPSS Statistics version 28 and GraphPad Prism version 10 [18, 19]. Analyses were performed per protocol: participants who had M0 and Y3 serum samples collected were included in the final analysis.

### Ethical Considerations

The study was conducted according to the principles of the Declaration of Helsinki and in accordance with the Medical Research Involving Human Subjects Act (WMO). In 2018, the research ethics committee of the Amsterdam UMC provided ethical clearance (NL65687.018.18); an amendment to add a 3 year follow-up moment was approved in 2021. The study was registered in the Dutch trial registry (number 7385). All participants provided a separate written informed consent for participating in the follow-up study.

## RESULTS

### Demographics

**Figure 1.**
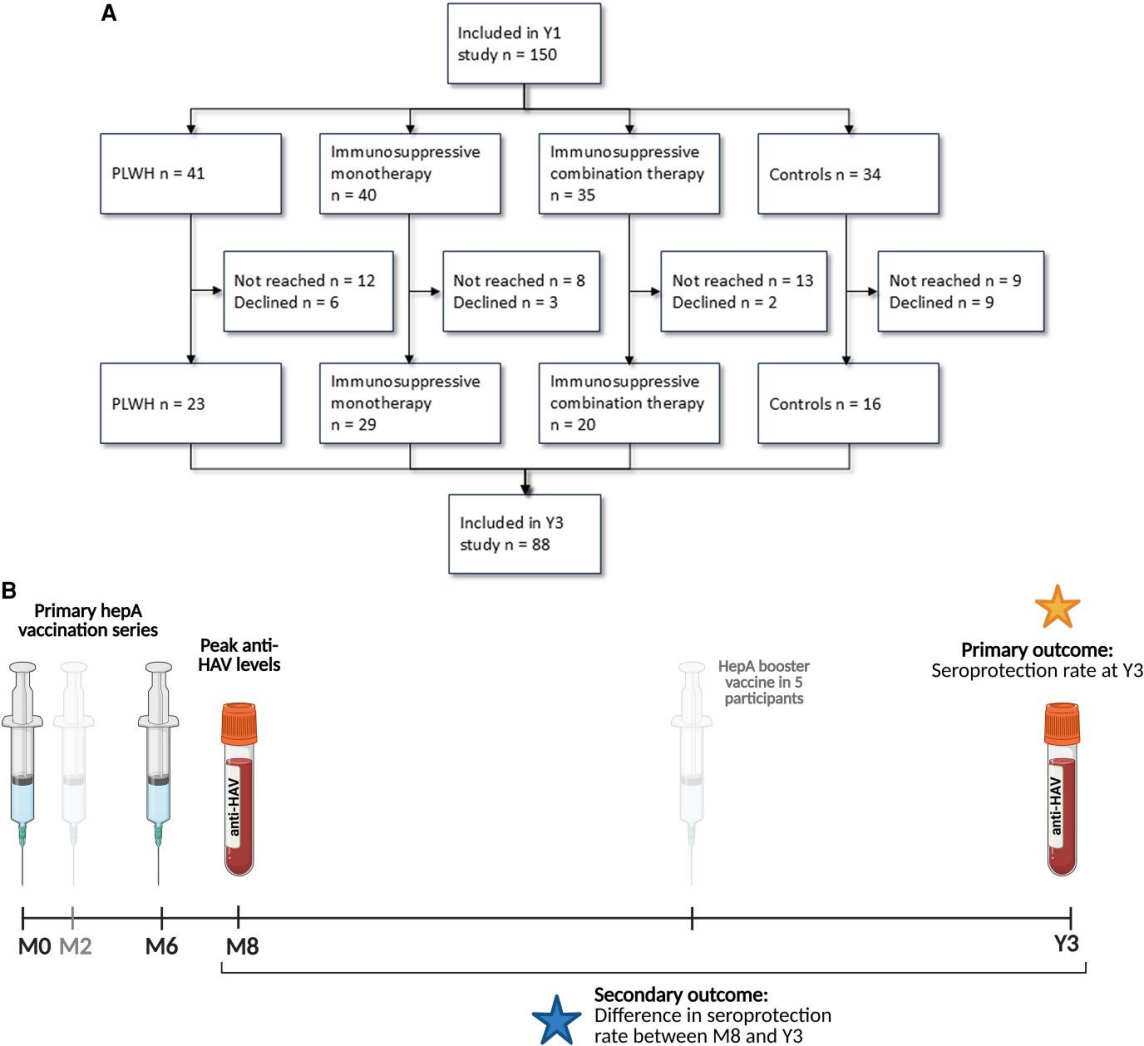
*A*, Study flowchart. *B*, Study design. Created in Biorender.com.

Of the initial 150 study participants, 88 (59%) individuals were enrolled in this follow-up study, including 23 PLWH, 29 patients on immunosuppressive monotherapy, 20 on immunosuppressive combination therapy, and 16 controls (Figure 1). Patients who were included in the 3-year follow-up study were significantly older (40.5 years [IQR 30–50.5]) than those who did not participate (34 years [IQR 24–47]) (*P*-value .022) (Supplementary Table 1). Twenty-two participants (25%) had received the combined hepA/B vaccine instead of the monovalent hepA vaccine. Five participants had received an additional hepA booster vaccination (third dose of the monovalent hepA vaccine) before the Y3 sample collection, who were all on immunosuppressive combination therapy. PLWH were all using cART, all had an undetectable viral load, and had a median CD4 count of 650 cells/mm^3^. The most common underlying diagnosis among patients on immunosuppressive therapy was inflammatory bowel disease (IBD) (30/49 [61%]). Of those patients on immunosuppressive monotherapy, 45% (13/29) used a conventional immunomodulator (cIM), and 55% (16/29) a biological immunomodulator (bIM). All patients on immunosuppressive combination therapy used a cIM (20/20), of whom 75% (15/20) used a biological as well (Table 1). One participant used B-cell depleting anti-CD20 therapy (rituximab), whose details are described separately, and who was excluded from the analysis (Supplementary Table 2).

**Table 1.** Baseline Characteristics of Study Participants

Overall (*n* = 88)	Total	PLWH	Immunosuppressive Therapy		Controls	*P-*value Across Groups
			Monotherapy	Combination		
Males, *n* (%)	52/88 (59%)	22/23 (96%)	13/29 (45%)	10/20 (50%)	7/16 (44%)	**<**.**001**
Age, median (IQR)	40.5 (30–50.5)	43 (35.5–52)	40 (32–50)	43.5 (24.5–51.5)	35 (28–45)	.288
BMI kg/m^2^, median (IQR)	23 (21–26)	24 (21–26.5)	23 (21–26)	23.5 (21.5–25.5)	23 (21–26.5)	.936
Charlson comorbidity index, median (IQR)	1 (0–1)	0 (0–1)^b^	1 (1–2)^a^	1 (1–2)^a^	0 (0–1)^b^	**<**.**001**
Impaired kidney function (eGFR <60), *n* (%)	8/88 (9%)	3/23 (13%)	2/29 (6%)	3/20 (15%)	0/16 (0%)	.384
Current or past smoker (ref: never smoked), *n* (%)	46/88 (52%)	14/23 (61%)	14/29 (48%)	9/20 (45%)	9/16 (56%)	.707
Heavy alcohol use (>21 units/wk for male and >14/units/wk for female), *n* (%)	4/88 (5%)	1/23 (4%)	2/29 (7%)	0/20 (0%)	1/16 (6%)	.757
Illicit drug use, *n* (%)	16/88 (18%)	11/23 (48%)	2/29 (7%)	1/20 (5%)	2/12 (13%)	**<**.**001**
Hepatitis A/B vaccine, *n* (%)	22/88 (25%)	9/23 (39%)	9/23 (31%)	2/20 (10%)	2/16 (13%)	.080
PCV13 coadministration, *n* (%)	49/88 (56%)	16/23 (70%)	20/29 (69%)	11/20 (55%)	2/16 (13%)	.**001**
DTP vaccine coadministration, *n* (%)	26/88 (30%)	2/23 (9%)	9/29 (31%)	4/20 (20%)	11/16 (69%)	**<**.**001**
Rabies vaccine coadministration, *n* (%)	12/88 (14%)	0/23 (0%)	10/29 (35%)	0/20 (0%)	2/16 (13%)	**<**.**001**
Hepatitis A booster vaccination, *n* (%)	5/88 (6%)	0/23 (0%)	0/29 (0%)	5/20 (25%)	0/16 (0%)	**<**.**001**
HIV-specific (*n* = 23)						
Time between HIV diagnosis and first hepA vaccine, years (median, IQR)	NA	4 (1–9.5)	NA	NA	NA	NA
AIDS at time of diagnosis, *n* (%)	NA	1/23 (4%)	NA	NA	NA	NA
cART use, *n* (%)	NA	23/23 (100%)	NA	NA	NA	NA
Undetectable viral load (<20 copies/mL), *n* (%)	NA	23/23 (100%)	NA	NA	NA	NA
Current CD4 count first hepA vaccine, cells/mm^3^ (median, IQR)	NA	650 (400–795)	NA	NA	NA	NA
Current CD4 count <500 cell/mm^3^, *n* (%)	NA	9/23 (39%)	NA	NA	NA	NA
Nadir CD4 count cells/mm^3^ (median, IQR)	NA	250 (79–360)	NA	NA	NA	NA
Nadir CD4 count <200 cells/mm^3^, *n* (%)	NA	7/21 (8%)	NA	NA	NA	NA
CD4/CD8 ration (median, IQR)	NA	0.64 (0.46–1.00)	NA	NA	NA	NA
Immunosuppressants-specific (*n* = 49)						
Underlying disease	…	…	…	…	…	.219
Inflammatory bowel disease, *n* (%)	NA	NA	15/29 (52%)	15/20 (75%)	NA	
Psoriasis/psoriatic arthritis, *n* (%)	NA	NA	5/29 (17%)	1/20 (5%)	NA	
Other diagnosis^d^, *n* (%)	NA	NA	9/29 (31%)	4/20 (20%)	NA	
Conventional immunomodulator	NA	NA	13/29 (45%)	20/20 (100%)	NA	**<.001**
Methotrexate (7.5–25 mg/wk)	NA	NA	4/29 (14%)	5/20 (25%)	NA	.319
Thiopurine^c^	NA	NA	7/29 (24%)	13/20 (65%)	NA	**.004**
Other^e^	NA	NA	2/29 (7%)	5/20 (25%)	NA	.075
Biological immunomodulator	NA	NA	16/29 (55%)	15/20 (75%)	NA	.157
Adalimumab (40 mg per 2 wks)	NA	NA	8/29 (28%)	3/20 (15%)	NA	.299
Infliximab (1–8.19 mg/kg per 4–8 wks)	NA	NA	5/29 (17%)	9/30 (45%)	NA	**.035**
Other^f^	NA	NA	3/29 (10%)	3/20 (15%)	NA	.625
Low-dose prednisolone (5–10 mg)	NA	NA	3/29 (10%)	3/20 (15%)	NA	.625

Significant *P*-values are in bold. *P*-values for dichotomous data were χ^2^, or by Fisher's Exact test when values in any of the cells were below 5; *P*-values of numerical data were calculated by Kruskal–Wallis test (data not normally distributed).

Abbreviations: PLWH, people living with HIV; BMI, body mass index; eGFR, estimated glomerular filtration rate; PCV13, Pneumococcal conjugate vaccine (Prevenar 13®); DTP, diphtheria, tetanus and polio vaccine; cART, combined antiretroviral therapy.

^a^Is higher than.

^b^Is higher than.

^c^Kidney transplantation (*n* = 3), rheumatoid arthritis (*n* = 3), autoimmune hepatitis (*n* = 1), spondyloarthritis (*n* = 1), sarcoidosis (*n* = 1), Behçet's disease (*n* = 1), juvenile idiopathic arthritis (*n* = 1), juvenile idiopathic arthritis and uveitis anterior (*n* = 1), membranoproliferative glomerulonephritis type 1 (*n* = 1).

^d^The (*n* = 4), azathioprine (50–200 mg) (*n* = 14); mercaptopurine (10–1000 mg) (*n* = 4), thioguanine (20 mg) (*n* = 4).

^e^Prednisolone (>10 mg/d or 700 mg cumulative) (*n* = 2), mycophenolate mofetil (*n* = 3), tacrolimus (*n* = 2), cyclosporine (*n* = 1), mycophenolic acid (*n* = 1), and hydroxyurea (*n* = 1).

^f^Ustekinumab (*n* = 4), secukinumab (*n* = 1), and rituximab (*n* = 1).

### Seroprotection Rates

**Figure 2.**
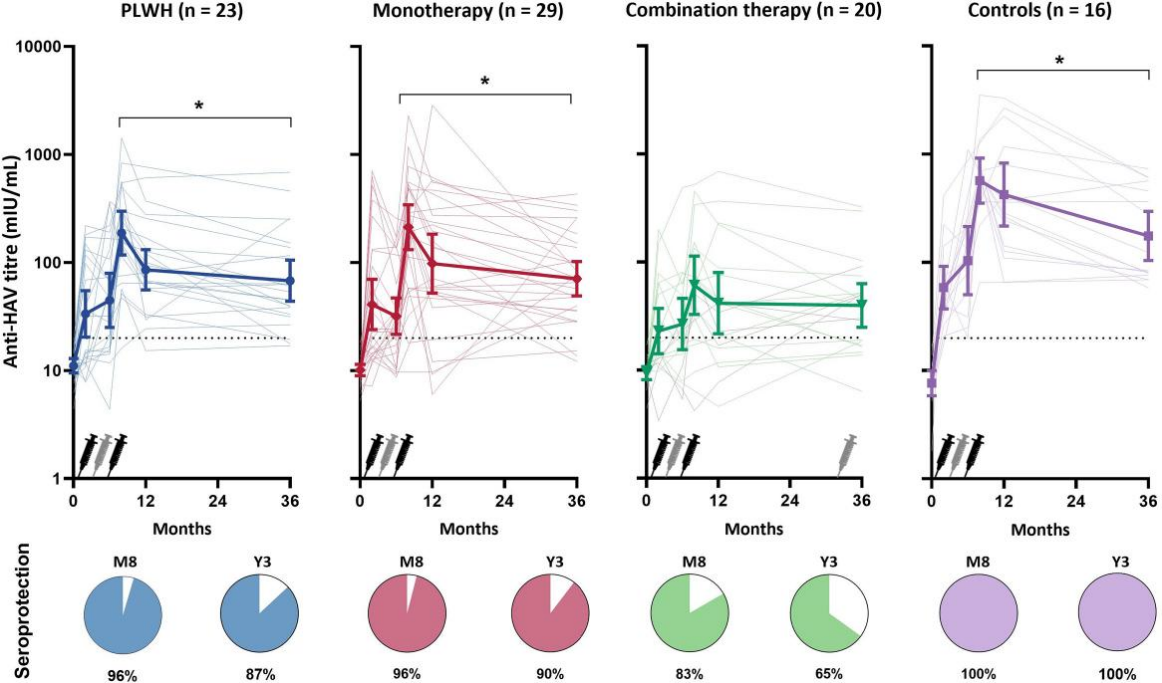
Dynamics of geometric mean anti-hepatitis A virus (HAV) concentrations up to 3 years after hepatitis A vaccination in immunocompromised populations (ICPs) and controls. The figures displays the serological responses among people living with HIV (PLWH), patients on immunosuppressive monotherapy, patients on immunosuppressive combinations and controls, who received a full hepA vaccination series (either 2 hepA vaccine doses at months 0 and 6–12, or 3 combined hepA/B vaccine doses at months 0, 1, and 6–12). The symbols depict geometric mean concentrations and 95% confidence intervals. The lighter colored lines depict anti-HAV concentrations of individual participants. Five patients on combination therapy received a hepA booster vaccination before Y3 measurement. Their individual anti-HAV concentrations are depicted in gray lines. The dotted line indicates an anti-HAV level of 20 mIU/mL. The charts below display seroconversion rates (proportion of participants with anti-HAV levels ≥20 mIU/mL). * In all groups median antibodies significantly declined between M8 and Y3.

**Figure 3.**
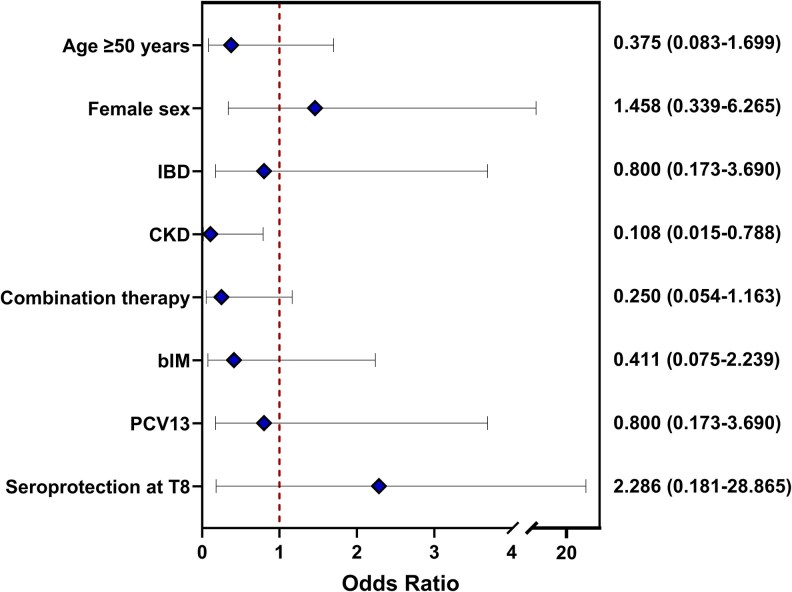
Factors associated with seroprotection at Y3 among patients on immunosuppressive therapy. Univariable logistic regression of variables of interest and seroprotection at Y3. Numbers on the right display odds ratios with 95% confidence intervals. Seroprotection rates stratified by these factors are provided in Supplementary Table 7. The sole patient on anti-CD20 therapy was left out of this analysis (for details see Supplementary Table 2). Abbreviations: IBD, inflammatory bowel disease; CKD, chronic kidney disease; bIM, biological immunomodulators. PCV13, Pneumococcal conjugate vaccine (Prevenar 13®).

From M8 (peak antibody levels) and Y3, SPRs showed a 10% decline among PLWH from 97% (33/34) to 87% (20/23), a 4% decline among patients on immunosuppressive monotherapy from 94% (32/34) to 90% (26/29), and an 18% decline among patients on immunosuppressive combination therapy from 83% (25/30) to 65% (13/20). By contrast, no decline was observed among controls, where SPRs remained 100% (16/16) (Figure 2). The decline in SPRs was not statistically significant in any of the groups (*P*-value = .092 for overall decline). At Y3, the SPR in patients on combination therapy was significantly lower than in controls (*P* = .011), especially among those who had not received an additional booster (Supplementary Tables 3 and 4). Among those, SPRs declined with 30% to 53% (8/15). Among those who were seroprotected at M8 and had a Y3 serum available, 13% (3/23) of PLWH, 10% (3/29) of patients on immunosuppressive monotherapy, 21% (4/19) of patients on immunosuppressive combination therapy and none (0/16) of the controls seroreverted at Y3. Seroreversion rates did not significantly differ between groups (*P*-value = .275). The patient on anti-CD20 therapy was not seroprotected at M8 nor at Y3. No logistic regression analysis was performed to identify predictors for seroprotection for PLWH, as only 3 participants were not seroprotected at Y3. Stratification by relevant variables displayed no notable trends (Supplementary Table 5). Univariable logistic regression among patients on immunosuppressive therapy showed an association with chronic kidney disease (OR 0.108; 95% CI: .015–.788) with lower SPRs at Y3 (Figure 3; Supplementary Table 6). No multivariable logistic regression was performed as only 10 patients on immunosuppressive therapy were not seroprotected at Y3. Of the 5 patients on immunosuppressive combination therapy who had received an additional booster vaccination, 1 was seroprotected at the day of booster vaccination, while all were seroprotected at Y3 (Supplementary Figure 3). Further details on the 13 participants who were not protected at Y3 are provided in Supplementary Table 7 and Supplementary Figure 4. None of the participants reported having experienced jaundice or a diagnosis of hepatitis A during the follow-up period.

### Geometric Mean Antibody Concentrations

At Y3, GMCs were 67.95 mIU/mL (95% CI: 43.78–105.46) for PLWH, 70.75 mIU/mL (95%CI: 49.06–102.02) for patients on immunosuppressive monotherapy, and 41.13 mIU/mL (95%CI: 25.27–66.93) mIU/mL for patients on immunosuppressive combination therapy, whereas these were 175.65 (95% CI: 104.37–295.60) mIU/mL for controls. GMCs were significantly lower in ICPs compared to controls (*P*-value = .001). GMCs significantly declined between M8 and Y3 for PLWH, patients on immunosuppressive monotherapy and controls (*P*-value = .001). This decline was also significant for patients on combination therapy, but only after exclusion of those who had received a booster vaccination between M24 and Y3 (*P*-value = .009) (Figure 2; Supplementary Tables 8 and 9). GMCs of the 5 patients who had received a booster vaccination were 28.75 (95% CI: 6.99–118.25) mIU/mL at M8 and 45.72 (95% CI: 25.52–81.92) mIU/mL at Y3. Median fold decreases in GMCs did not significantly differ between groups (*P*-value = .113). No significant predictors were found for the decline in GMCs between M8 and Y3 among PLWH with univariable linear regression. No multivariable linear regression was performed because of the small sample size of PLWH (Supplementary Table 10). Among patients on immunosuppressive therapy, use of combination therapy (β = 0.286 [95%CI: .001–.571]) and having received a hepA booster vaccination (β = 0.633 [95% CI: .226–1.039]) were associated with a smaller decrease of GMCs between M8 and Y3, whereas higher anti-HAV levels at M8 were associated with a bigger decrease (β = −0.509 [95% CI: −.711–−.307]). After adjusting for booster vaccination, the association with combination therapy lost statistical significance (β = 0.125 [95% CI: −.176–.426]). Associations for use of combination therapy and booster vaccination did not remain significant after adjustment for age, sex, and anti-HAV levels at M8, while anti-HAV at M8 did remain significantly associated in the multivariable models (Supplementary Table 11).

## DISCUSSION

The majority of PLWH, patients on immunosuppressive monotherapy and patients on combination therapy were seroprotected 3 years after a complete hepA vaccination series (87%, 90%, 65%, respectively), compared to 100% of immunocompetent controls. We found that hepA antibody concentrations declined at a similar rate in ICPs and controls after excluding those who received a booster. This suggests that the lower SPRs in ICPs are because of lower peak antibody levels postvaccination rather than accelerated waning. Among initial responders to vaccination, 13% of PLWH, 10% of patients on immunosuppressive monotherapy, and 21% of patients on immunosuppressive combination therapy lost their serologic protection 3 years after vaccination. For PLWH, our findings are in line with 2 studies that were conducted in previous, less immunocompetent PLWH cohorts. First, Crum–Cianflone *et al.* reported a seroreversion rate of 10% after 3 years in PLWH who were initial responders (mean CD4 461, 62% cART coverage) [10]. Cheng *et al.*, found that 88% of participants were still protected 5 years after a 2-dose vaccination regimen (mean CD4 485, 62% cART coverage) [11]. With regard to immunosuppressive therapy, our data correspond to those of Askling *et al.*, who found that 14% of patients with rheumatoid arthritis (RA) treated with tumor necrosis factor inhibitors and/or methotrexate who were initially seroprotected, had seroreverted 2 years after a 2-dose hepA vaccination series [16]. In our cohort, SPRs were lowest among those on immunosuppressive combination therapy, and chronic kidney failure was associated with lower SPRs at Y3. This aligns with an earlier study showing lower SPR 2 years postvaccination in renal transplant recipients mostly on immunosuppressive combination therapy compared with liver transplant recipients, who mostly received immunosuppressive monotherapy (6/23 [26%] vs 16/27 [59%]). The lower SPRs in that study may be explained by their higher seroprotection threshold (≥33 mIU/mL) [20].

### Limitations

One limitation of this follow-up study was the substantial loss to follow-up, despite extensive efforts to contact and recruit initial participants, which reduced the sample size. This may have limited the possibility to detect differences between groups and to identify potential predictors of long-term seroprotection. Additionally, those who did participate in this follow-up study were significantly older than those who did not (40.5 vs 34 years, respectively), which may have led to an underestimation of protection at Y3. Another limitation of this study was that the long-term protection was assessed by correlates of immunity. Large sample sizes are needed to establish clinical efficacy of hepA vaccination, which is not feasible for ICPs. Commonly, a serological cutoff for seroprotection of anti-HAV ≥10 or 20 mIU/mL is applied, corresponding to the lower limit of detection of the particular assay being used [6]. This cutoff was established by dose-response studies in passively immunized chimpanzees who were challenged with virulent HAV [21], as well as by efficacy studies in children [22, 23]. Furthermore, this study was limited to the measurement of humoral responses, while vaccine-induced memory B- and T-cells also contribute to long-term protection against disease [7, 24].

### Recommendations for Clinical Practice

Our findings, along with previous studies, suggest that humoral seroprotection after hepA vaccination is less durable in PLWH and patients on immunosuppressive therapy compared to controls. However, vaccine-induced immunological memory may persist longer than detectable antibody levels in peripheral blood in ICPs. During the initial study period, all PLWH and patients on immunosuppressive monotherapy with impeded antibody levels achieved seroprotection within 7 days after booster vaccination 1 to 5 years after primary vaccination, indicating intact immunological memory. By contrast, only half of patients on immunosuppressive combination therapy were boostable [8]. This raises the question whether revaccination is actually needed for PLWH and patients on immunosuppressive monotherapy who seroreverted after initially mounting a protective response, similar to hepatitis B vaccination guidelines. This is further supported by a recent systematic review that found that hepA breakthrough infections are rare, but more common in ICPs who did not seroconvert after vaccination, whereas no symptomatic breakthrough infections were reported in initial responders [25]. As PLWH and patients on immunosuppressive monotherapy who initially responded to primary vaccination may retain long-term protection through immunological memory, routine antibody measurements, and revaccination prior to travel may be unnecessary in these individuals. However, in patients on immunosuppressive combination therapy, the impaired ability to generate an adequate booster response suggests that monitoring antibody levels with a booster-on-demand remains necessary to ensure sustained protection against hepA. All 5 patients on immunosuppressive combination therapy who had received a booster vaccination in our cohort, achieved antibodies at Y3 that were similar or even higher compared to M8. Thus, we anticipate that the majority of patients on immunosuppressive combination therapy will seroconvert 1 month after booster vaccination. Those who do not seroconvert may receive hepA immunoglobulins prior to travelling.

### Conclusions

Three years postvaccination, most PLWH and patients on immunosuppressive therapy remained seroprotected, but SPRs and GMCs were lower than in healthy controls, particularly among those on combination therapy. These findings, based on a small and heterogeneous cohort, suggest that serological responses mounted by the current hepA vaccination series are less durable among ICPs. However, it remains uncertain if booster doses are necessary among those with a good initial postvaccination peak antibody level, as long-term protection may persist through formed cellular memory.

## Supplementary Material

ofaf457_Supplementary_Data

## Supplementary Data

Supplementary materials are available at *Open Forum Infectious Diseases* online. Consisting of data provided by the authors to benefit the reader, the posted materials are not copyedited and are the sole responsibility of the authors, so questions or comments should be addressed to the corresponding author.
