# Supplementary material for: Long-term Immunogenicity of Hepatitis A Vaccination in Adults Receiving Immunosuppressive Therapy and Adults Living With HIV: Three-year Follow-up of a Prospective Cohort Study
Source: Open Forum Infect Dis. 2025 Sep 15;12(9):ofaf457. doi: 10.1093/ofid/ofaf457 (PMC12448379; doi:10.1093/ofid/ofaf457)
Supplement: ofaf457_Supplementary_Data [file ofaf457_supplementary_data.docx]

**Supplementary Material**

**Table of contents**

[**Supplementary Table 1.** Demographics of all participants who were included in the year 1 study (n = 150): comparison of those participating in the three year follow up versus those not participating. 1](#_Toc195515999)

[**Supplementary Table 2.** Details of the participant on anti-CD20 therapy. 5](#_Toc195516000)

[**Supplementary Table 3.** Seroprotection rates across groups. 6](#_Toc195516001)

[**Supplementary Table 4.** Seroprotection rates at Y3 among patients on combination therapy with and without booster vaccination. 7](#_Toc195516002)

[**Supplementary Table 5.** Seroprotection rates at Y3 stratified by relevant variables among PLWH. 8](#_Toc195516003)

[**Supplementary Table 6.** Seroprotection rates at Y3 stratified by relevant variables among patients on immunosuppressive therapy. 9](#_Toc195516004)

[**Supplementary Table 7.** Details on the 13 participants who were not seroprotected at Y3. 10](#_Toc195516005)

[**Supplementary Table 8.** Geometric mean antibody concentrations (GMC). 11](#_Toc195516006)

[**Supplementary Table 9.** Geometric mean antibody concentrations (GMC) without participants who received a booster vaccination. 12](#_Toc195516007)

[**Supplementary Table 10**. Univariable linear regression among people living with HIV. 13](#_Toc195516008)

[**Supplementary Table 11**. Univariable and multivariable linear regression among patients on immunosuppressive therapy. 14](#_Toc195516009)

[**Supplementary Figure 1.** Directed acyclic graph (DAG) representing the relationships between variables of interest in the analysis of hepatitis A vaccination responses in people living with HIV (PLWH) based on findings from the literature. 15](#_Toc195516010)

[**Supplementary Figure 2.** Directed acyclic graph (DAG) representing the relationships between variables of interest in the analysis of hepatitis A vaccination responses in patients on immunosuppressive therapy based on findings from the literature. 16](#_Toc195516011)

[**Supplementary Figure 3.** Anti-HAV levels over time of the five patients on combination therapy who received an additional booster vaccine dose. 17](#_Toc195516012)

[**Supplementary Figure 4.** Anti-HAV levels over time of the thirteen participants who were not seroprotected at Y3. 18](#_Toc195516013)

**Supplementary Table 1.** Demographics of all participants who were included in the year 1 study (n = 150): comparison of those participating in the three year follow up versus those not participating.

| Total (n = 150) | Included (n = 88) | Not included (n = 62) | *p*-value |
| --- | --- | --- | --- |
| Males, *n* (%) | 52/88 (59%) | 32/62 (52%) | 0.364 |
| Age, median (IQR) | 40.5 (30-50.5) | 34 (24-47) | **0.022** |
| BMI kg/m2, median (IQR) | 23 (21-26) | 24 (21-26) | 0.841 |
| Charlson co-morbidity index, median (IQR) | 1 (0-1) | 1 (0-1) | 0.838 |
| Impaired kidney function (eGFR <60), *n* (%) | 8/88 (9%) | 6/62 (10%) | 0.903 |
| Current or past smoker (ref: never smoked), *n* (%) | 46/88 (52%) | 28/62 (45%) | 0.391 |
| Heavy alcohol use (>21 units/week for male and >14/units/week for female), *n* (%) | 4/88 (5%) | 0/62 (0%) | 0.137 |
| Illicit drug use, *n* (%) | 16/88 (18%) | 10/62 (16%) | 0.744 |
| HepA booster vaccination, *n* (%) | 5/88 (6%) | 3/62 (5%) | 1.00 |
| Hepatitis A/B vaccine, *n* (%) | 22/88 (25%) | 9/62 (15%) | 0.118 |
| PCV13 co-administration, *n* (%) | 49/88 (56%) | 25/62 (40%) | 0.064 |
| DTP vaccine co-administration, *n* (%) | 26/88 (30%) | 21/62 (34%) | 0.574 |
| Rabies vaccine co-administration, *n* (%) | 12/88 (14%) | 3/62 (5%) | 0.099 |
| PLWH (n = 41) | **Included (n =23)** | **Not included (n = 18)** | ***p*-value** |
| Time between HIV diagnosis and first hepA vaccine, years (median, IQR) | 4 (1-9.5) | 9 (7-16) | **0.019** |
| AIDS at time of diagnosis, *n* (%) | 1/23 (4%) | 0/18 (0%) | 0.561 |
| cART use, *n* (%) | 23/23 (100%) | 18/18 (100%) | NA |
| Undetectable viral load (<20 copies/ml), *n* (%) | 23/23 (100%) | 18/18 (100%) | NA |
| CD4 count at first hepA vaccine, cells/mm^3^ (median, IQR) | 650 (400-795) | 715 (560-870) | 0.511 |
| CD4 count <500 cell/mm^3^, *n* (%) | 9/23 (39%) | 4/18 (22%) | 0.321 |
| Nadir CD4 count cells/mm^3^ (median, IQR) | 250 (79-360) | 290 (165-500) | 0.446 |
| Nadir CD4 count <200 cells/mm^3^, *n* (%) | 7/21 (33%) | 5/15 (33%) | 1.00 |
| CD4/CD8 ratio (median, IQR) | 0.64 (0.46-1.00) | 1.1 (0.79-1.24) | **0.008** |
| Immunosuppressive monotherapy (n = 40) | **Included (n = 29)** | **Not included (n = 11)** | ***p*-value** |
| *Underlying disease*  Inflammatory bowel disease, *n* (%)  Psoriasis/psoriatic arthritis, *n* (%)  Other diagnosis, *n* (%) | 15/29 (52%)  5/29 (17%)  9/29 (31%) | 7/11 (64%)  0/11 (11%)  4/11(36%) | 0.338 |
| *Conventional immunomodulator*  Methotrexate (7.5-25 mg/week)  Thiopurine  Other | 13/29 (45%)  4/29 (14%)  7/29 (24%)  2/29 (7%) | 4/11 (36%)  2/11 (18%)  2/11 (18%)  0/11 (0%) | 0.730  1.00  1.00  1.00 |
| *Biological immunomodulator*  Adalimumab (40 mg per 2 weeks)  Infliximab (1-8.19 mg/kg per 4-8 weeks)  Other | 16/29 (55%)  8/29 (28%)  5/29 (17%)  3/29 (10%) | 7/11 (64%)  2/11 (18%)  2/11 (18%)  3/11 (27%) | 0.730  0.696  1.00  0.319 |
| Low-dose prednisolone (5-10 mg) | 3/29 (10%) | 0/11 (0%) | 0.548 |
| Immunosuppressive combination therapy (n = 35) | **Included (n = 20)** | **Not included (n = 15)** | ***p*-value** |
| *Underlying disease*  Inflammatory bowel disease, *n* (%)  Psoriasis/psoriatic arthritis, *n* (%)  Other diagnosis§, *n* (%) | 15/20 (75%)  1/20 (5%)  4/20 (20%) | 10/15 (67%)  0/15 (0%)  5/15 (3%) | 0.490 |
| *Conventional immunomodulator*  Methotrexate (7.5-25 mg/week)  Thiopurine  Other | 20/20 (100%)  5/20 (25%)  13/20 (65%)  5/20 (25%) | 13/15 (87%)  2/15 (13%)  7/15 (47%)  5/15 (33%) | 0.176  0.672  0.278  0.712 |
| *Biological immunomodulator*  Adalimumab (40 mg per 2 weeks)  Infliximab (1-8.19 mg/kg per 4-8 weeks)  Other | 15/20 (75%)  3/20 (15%)  9/30 (45%)  3/20 (15%) | 12/15 (80%)  4/15 (27%)  8/15 (53%)  1/15 (7%) | 1.00  0.430  0.625  0.619 |
| Low-dose prednisolone (5-10 mg) | 3/20 (15%) | 1/15 (7%) | 0.619 |

Significant *p*-values are in **bold**. *p*-values for dichotomous data were Chi-square, or by Fisher’s Exact Test when values in any of the cells were below 5; *p*-values of numerical data were calculated by independent samples Mann-Whitney U test (data not normally distributed). PLWH = people living with HIV; IQR = interquartile range; NA = not applicable.

**Supplementary Table 2.** Details of the participant on anti-CD20 therapy.

| Age | Sex | Group | Underlying diagnosis | Immunosuppressive therapy |
| --- | --- | --- | --- | --- |
| 45 | Female | Immunosuppressive combination therapy | Rheumatoid arthritis | Methotrexate 12.5 mg every week, initiated 14 years before vaccination  Rituximab 500 mg every 6 months, initiated three years before vaccination (last dose 1.5 months prior to vaccination) |

**Supplementary Table 3.** Seroprotection rates across groups.

|  | PLWH | Immunosuppressive monotherapy | Immunosuppressive combination therapy | Controls | *p*-value difference between groups |
| --- | --- | --- | --- | --- | --- |
| SCR at M8 | 21/22 (96%) | 24/25 (96%) | 15/18 (94%) | 15/15 (100%) | 0.199 |
| SCR at Y3 | 20/23 (87%) | 26/29 (90%) | 13/20 (65%)† | 16/16 (100%)* | **0.021** |
| *p*-value difference between M8 and Y3 | 0.625 | 0.625 | 0.375 | NA | NA |

Significant *p*-values are in **bold**. *p*-values comparing difference in SPR between M8 and Y3 were calculated by the McNemar test. *p*-values comparing difference between groups were calculated by Fisher’s Exact Test and adjusted by Bonferroni correction for multiple tests. Overall difference between M8 and Y3 was also not significant (*p*-value = 0.092). * Is significantly higher than † (*p*-value 0.011). SCR = seroconversion rate; PLWH = people living with HIV.

**Supplementary Table 4.** Seroprotection rates at Y3 among patients on combination therapy with and without booster vaccination.

|  | Overall  (% [95%CI]) | Without booster  (% [95%CI]) | With booster  (% [95%CI]) |
| --- | --- | --- | --- |
| Overall | 75/88 (85% [77-92%]) | 70/83 (84% [75-91%]) | 5/5 (100% [62-100%] |
| Patients on combination therapy | 13/20 (65% [43-83%]) | 8/15 (53% [29-76%]) | 5/5 (100% [62-100%] |

**Supplementary Table 5.** Seroprotection rates at Y3 stratified by relevant variables among PLWH.

|  | **SPR at Y3** | **Odds ratio (95%CI)** | ***p*-value** |
| --- | --- | --- | --- |
| **Age, *n* (%)** |  |  |  |
| 18-49 years | 14/15 (93%) |  |  |
| ≥ 50 years | 6/8 (75%) | 0.214 (0.016-2.839) | 0.269 |
| **Sex, *n* (%)** |  |  |  |
| Male | 19/22 (86%) |  |  |
| Female | 1/1 (100%) | NE | 1.000 |
| **Time since HIV-diagnosis, *n* (%)** |  |  |  |
| < 10 months | 14/17 (82%) |  |  |
| ≥ 10 months | 6/6 (100%) | NE | 0.539 |
| **AIDS at diagnosis, *n* (%)** |  |  |  |
| No | 19/22 (86%) |  |  |
| Yes | 1/1 (100%) | NE | 1.000 |
| **CD4+ T cell number at M0, cells/mm^3^, *n* (%)** |  |  |  |
| <500 | 8/9 (89%) |  |  |
| ≥500 | 12/14 (86%) | 0.750 (0.058-9.719) | 1.00 |
| **Nadir CD4+ T cell number, cells/mm^3^, *n* (%)** |  |  |  |
| <200 | 7/7 (100%) |  |  |
| ≥200 | 11/14 (79%) | NE | 0.521 |
| **PCV13 co-administration, *n* (%)** |  |  |  |
| No | 6/7 (86%) |  |  |
| Yes | 14/16 (88%) | 1.167 (0.88-15.458) | 1.000 |
| **Seroprotection at M8, *n* (%)** |  |  |  |
| No | 1/1 (100%) |  |  |
| Yes | 18/21 (86%) | NE | 1.000 |

*p*-values were calculated by Fisher’s Exact Test. PCV13 = Pneumococcal conjugate vaccine (Prevenar 13®); NE = not estimated.

**Supplementary Table 6.** Seroprotection rates at Y3 stratified by relevant variables among patients on immunosuppressive therapy.

|  | **SPR at Y3** | **Odds ratio (95% CI)** | ***p*-value** |
| --- | --- | --- | --- |
| **Age, *n* (%)** |  |  |  |
| 18-49 years | 30/35 (86%) |  |  |
| ≥ 50 years | 9/13 (69%) | 0.375 (0.083-1.699) | 0.228 |
| **Sex, *n* (%)** |  |  |  |
| Male | 18/23 (78%) |  |  |
| Female | 21/25 (84%) | 1.458 (0.339-6.265) | 0.719 |
| **Underlying disease, *n* (%)** |  |  |  |
| Other than inflammatory bowel disease | 15/18 (83%) |  |  |
| Inflammatory bowel disease | 24/30 (80%) | 0.800 (0.173-3.690) | 1.000 |
| **Chronic kidney disease, *n* (%)** |  |  |  |
| No | 37/43 (86%) |  |  |
| Yes | 2/5 (40%) | 0.108 (0.015-0.788) | **0.039** |
| **Number of immunosuppressants, *n* (%)** |  |  |  |
| Monotherapy | 26/29 (90%) |  |  |
| Combination therapy | 13/19 (68%) | 0.250 (0.054-1.163) | 0.127 |
| **Type of immunosuppressive therapy, *n* (%)** |  |  |  |
| Biological immunomodulators | 16/18 (89%) |  |  |
| No biological immunomodulator | 23/30 (77%) | 0.411 (0.075-2.239) | 0.451 |
| **PCV13 co-administration, *n* (%)** |  |  |  |
| No | 15/18 (83%) |  |  |
| Yes | 24/30 (80%) | 0.800 (0.173-3.690) | 1.000 |
| **Seroprotection rates at M8, *n* (%)** |  |  |  |
| No | 2/3 (67%) |  |  |
| Yes | 32/39 (82%) | 2.286 (0.181-28.865) | 0.479 |
| **Hepatitis booster vaccination, *n* (%)** |  |  |  |
| No | 34/43 (79%) |  |  |
| Yes | 5/5 (100%) | NE | 0.568 |

Significant *p*-values are in **bold**. *p*-values were calculated by Fisher’s Exact Test. The patient on anti-CD20 therapy was left out of this analysis. PCV13 = Pneumococcal conjugate vaccine (Prevenar 13®); NE = not estimated.

**Supplementary Table 7.** Details on the 13 participants who were not seroprotected at Y3.

| Age | Sex | Group | CD4 count | Nadir CD4 count | | CD4/CD8 ratio | | HIV viral load |
| --- | --- | --- | --- | --- | --- | --- | --- | --- |
| 21 | M | PLWH | 770 | 400 | | 1.15 | | <20 copies/mL |
| 60 | M | PLWH | 430 | 240 | | 0.72 | | <20 copies/mL |
| 55 | M | PLWH | 780 | 340 | | 0.48 | | <20 copies/mL |
| Age | **Sex** | **Group** | | | **Underlying disease** | | **Immunosuppressive therapy** | |
| 54 | F | Immunosuppressive combination therapy | | | Kidney transplantation | | Tacrolimus + mycophenolate Mofetil | |
| 26 | M | Immunosuppressive combination therapy | | | IBD | | Infliximab + thioguanine | |
| 41 | F | Immunosuppressive combination therapy | | | Kidney transplantation | | Tacrolimus + azathioprine | |
| 45 | F | Immunosuppressive combination therapy | | | Rheumatoid arthritis | | Rituximab + methotrexate | |
| 49 | F | Immunosuppressive combination therapy | | | IBD | | Infliximab + methotrexate | |
| 62 | M | Immunosuppressive monotherapy | | | IBD | | Adalimumab | |
| 39 | M | Immunosuppressive monotherapy | | | IBD | | Infliximab | |
| 22 | F | Immunosuppressive combination therapy | | | IBD | | Infliximab + azathioprine | |
| 52 | M | Immunosuppressive monotherapy | | | Psoriasis | | Ustekinumab | |
| 65 | 1,00 | Immunosuppressive combination therapy | | | IBD | | Infliximab + mercaptopurine | |

F = female; M = male; IBD = inflammatory bowel disease.

**Supplementary Table 8.** Geometric mean antibody concentrations (GMC).

|  | PLWH | Immunosuppressive monotherapy | Immunosuppressive combination therapy | Controls | *p*-value difference between groups |
| --- | --- | --- | --- | --- | --- |
| GMC (95% CI) at M8 | 187.43 (117.76-298.33)†‡ | 212.39 (131.71-342.48)†‡ | 65.17 (34.19-124.23)†§ | 568.89 (352.50-918.12)* | **0.001** |
| GMC (95% CI) at Y3 | 67.95 (43.78-105.46)† | 70.75 (49.06-102.02)† | 41.13 (25.27-66.93)† | 175.65 (104.37-295.60)* | **0.001** |
| Median fold change in GMCs | 0.368 (0.109-0.93) | 0.276 (0.174-0.695) | 0.620 (0.286-1.392) | 0.173 (0.112-0.907) | 0.113 |
| *p*-value difference between M8 and Y3 | **<0.001** | **<0.001** | 0.138 | **<0.001** | NA |

Significant *p*-values are in **bold**. *p*-values for difference between M8 and Y3 were calculated by ANOVA test. *p*-values for difference between groups were calculated by ANOVA and adjusted by Bonferroni correction for multiple tests. * Is significantly higher than †, and ‡ is significantly higher than §. The patient on anti-CD20 therapy was left out of this analysis. CI = confidence interval; PLWH = people living with HIV; NA = not applicable.

**Supplementary Table 9.** Geometric mean antibody concentrations (GMC) without participants who received a booster vaccination.

|  | PLWH | Immunosuppressive monotherapy | Immunosuppressive combination therapy | Controls | *p*-value difference between groups |
| --- | --- | --- | --- | --- | --- |
| GMC (95% CI) at M8 | 187.43 (117.76-298.33)† | 212.39 (131.71-342.48)† | 91.65 (43.37-193.68)† | 568.89 (352.50-918.12)* | **<0.001** |
| GMC (95% CI) at Y3 | 67.95 (43.78-105.46)† | 70.75 (49.06-102.02)† | 39.60 (20.30-77.26)† | 175.65 (104.37-295.60)* | **<0.001** |
| Median fold change in GMCs | 0.368 (0.109-0.93) | 0.276 (0.174-0.695) | 0.501 (0.228-0.810) | 0.173 (0.112-0.907) | 0.829 |
| *p*-value difference between M8 and Y3 | **<0.001** | **<0.001** | **0.009** | **<0.001** | NA |

Significant *p*-values are in **bold**. *p*-values for difference between M8 and Y3 were calculated by ANOVA test. *p*-values for difference between groups were calculated by ANOVA and adjusted by Bonferroni correction for multiple tests. * Is significantly higher than †, and ‡ is significantly higher than §. The patient on anti-CD20 therapy and patients who received a booster vaccination were left out of this analysis. CI = confidence interval; PLWH = people living with HIV; NA = not applicable.

**Supplementary Table 10**. Univariable linear regression among people living with HIV.

| Variable | β (95%CI) | *p*-value |
| --- | --- | --- |
| Age | -0.003 (-0.017-0.011) | 0.666 |
| Sex | -0.288 (-1.019-0.444) | 0.422 |
| Time since HIV diagnosis (months) | -0.011 (-0.033-0.012) | 0.332 |
| AIDS diagnosis (ref: no AIDS) | -0.071 (-0.814-0.672) | 0.844 |
| CD4 count | 0.000 (-0.001-0.000) | 0.439 |
| Nadir CD4 count | -0.000 (-0.001-0.001) | 0.851 |
| PCV13 co-administration (ref: no PCV13) | 0.080 (-0.219-0.378) | 0.538 |
| Anti-HAV levels at M8 | -0.293 (-0.613-0.028) | 0.071 |

**Supplementary Table 11**. Univariable and multivariable linear regression among patients on immunosuppressive therapy.

|  | Univariable analysis | | Multivariable analysis | | | | | | | |
| --- | --- | --- | --- | --- | --- | --- | --- | --- | --- | --- |
| Variable | **β (95%CI)** | ***p*-value** | **Adjusted β (95%CI)** | ***p*-value** | **Adjusted β (95%CI)** | ***p*-value** | **Adjusted β (95%CI)** | ***p*-value** | **Adjusted β (95%CI)** | ***p*-value** |
| Age | -0.002 (-0.012-0.009) | 0.734 | -0.004 (-0.013-0.004) | 0.288 | -0.005 (-0.014-0.004) | 0.251 | -0.001 (-0.011-0.009) | 0.810 | NE | NE |
| Female (ref: male) | -0.142 (-0.432-0.149) | 0.331 | 0.058 (-0.181-0.297) | 0.626 | 0.039 (-0.206-0.285) | 0.748 | -0.047 (-0.326-0.231) | 0.733 | NE | NE |
| IBD (ref: other diagnosis) | -0,093 (-0,410-0,223) | 0.555 | NE | NE | NE | NE | NE | NE | NE | NE |
| CKD (ref: normal kidney function) | -0.235 (-0.731-0.260) | 0.342 | NE | NE | NE | NE | NE | NE | NE | NE |
| Combination therapy (ref: monotherapy) | 0.286 (0.001-0.571) | **0.049** | NE | NE | 0.010 (-0.258-0.279) | 0.938 | 0.120 (-0.189-0.430) | 0.436 | 0.125 (-0.176-0.426) | 0.406 |
| bIM (ref: no bIM) | -0.239 (-0.536-0.059) | 0.113 | NE | NE | NE | NE | NE | NE | NE | NE |
| PCV13 co-administration (ref: no PCV13) | 0.080 (-0.219-0.378) | 0.593 | NE | NE | NE | NE | NE | NE | NE | NE |
| Booster vaccination (ref: no booster) | 0.633 (0.226-1.039) | **0.003** | 0.297 (-0.095-0.689) | 0.133 | NE | NE | 0.534 (0.058-1.009) | **0.029** | 0.548 (0.092-1.004) | **0.020** |
| Anti-HAV levels at M8 | -0.509 (-0.711- -0.307) | **0.000** | -0.467 (-0.699- -0.236) | **0.000** | -0.533 (-0.77- -0.289) | **0.000** | NE | NE | NE | NE |

Significant *p*-values are in **bold**. The patient on anti-CD20 therapy was not included in this analysis; details for that particular patients are provided in **Supplementary Table 2**). NE = not estimated.

**Supplementary Figure 1.** Directed acyclic graph (DAG) representing the relationships between variables of interest in the analysis of hepatitis A vaccination responses in people living with HIV (PLWH) based on findings from the literature.


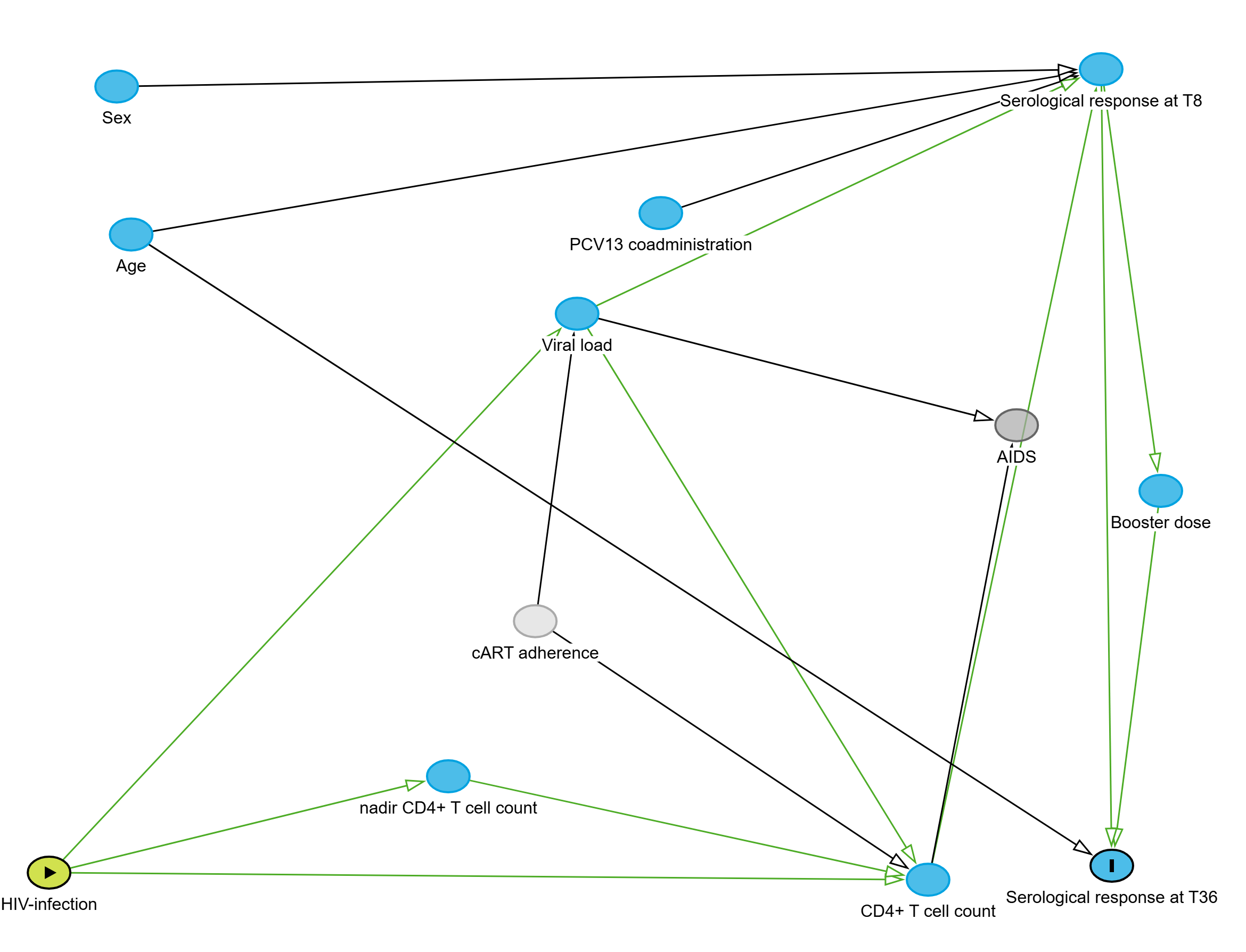


Blue circles represent ancestors of the outcome, thus may have a direct or indirect effect on serological responses at Y3. Light grey circles indicate variables that were not measured in our study cohort (unobserved variables).

**Supplementary Figure 2.** Directed acyclic graph (DAG) representing the relationships between variables of interest in the analysis of hepatitis A vaccination responses in patients on immunosuppressive therapy based on findings from the literature.


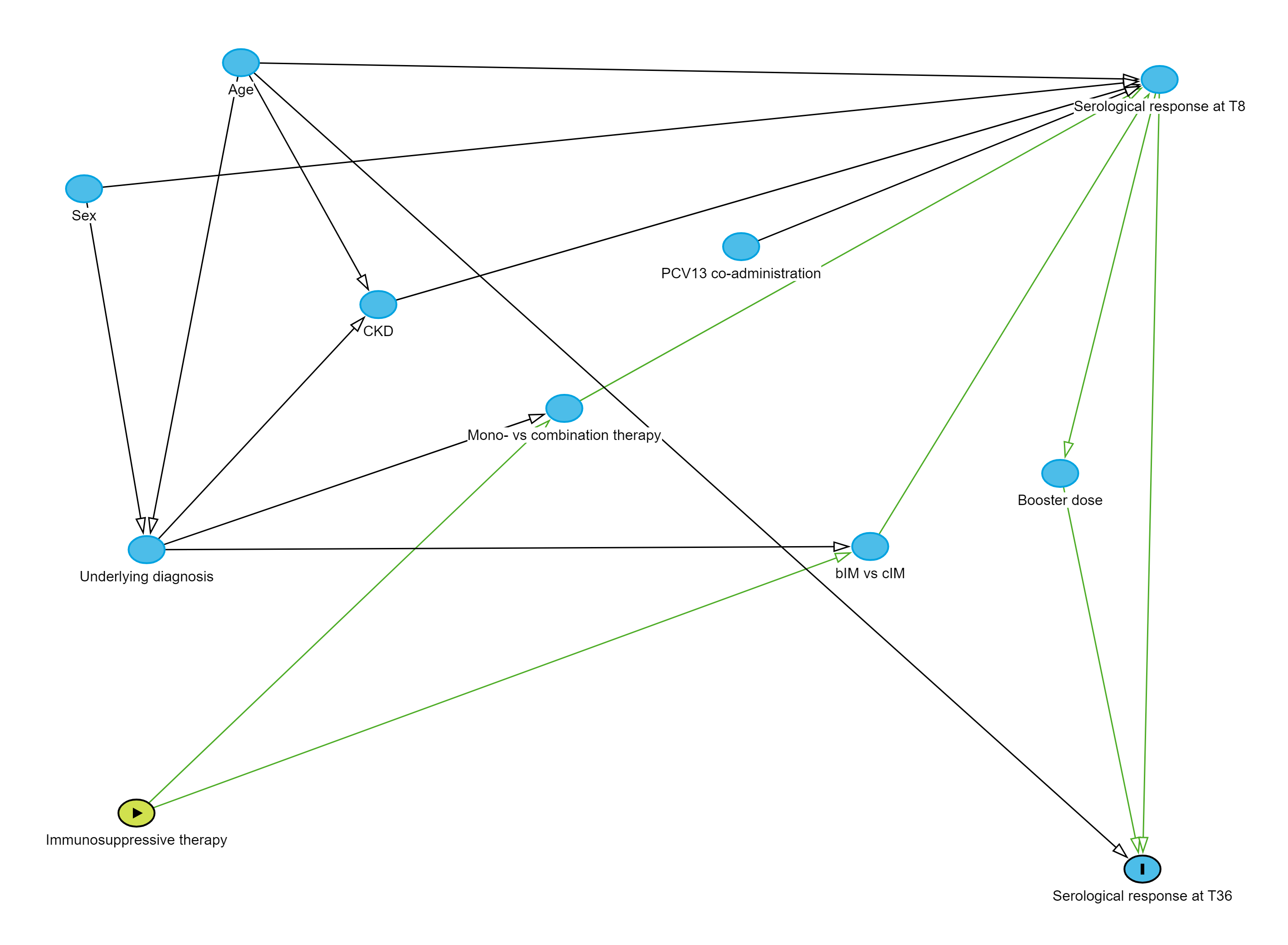


**Supplementary Figure 3.** Anti-HAV levels over time of the five patients on combination therapy who received an additional booster vaccine dose.

**Supplementary Figure 4.** Anti-HAV levels over time of the thirteen participants who were not seroprotected at Y3.
